# Cross-sectional Evaluation of Neurotology Fellowship Directors: A Present-day Snapshot of Leadership

**DOI:** 10.1097/ONO.0000000000000036

**Published:** 2023-07-27

**Authors:** Haley Hullfish, Benjamin Schachner, Zachary Zippi, Brandon Kamrava, Simon Angeli

**Affiliations:** 1 Department of Otolaryngology, Medical University of South Carolina, Charleston, SC; 2 Department of Otolaryngology, University of Miami Miller School of Medicine, Miami, FL; 3 Department of Education, Florida International University Herbert Wertheim College of Medicine, Miami, FL.

## Abstract

**Objective:**

To identify demographic, training, and career trends of neurotology fellowship directors (FDs).

**Study Design:**

Cross-sectional study.

**Setting:**

United States.

**Subjects:**

All 26 neurotology FDs identified using the American Neurotology Society (ANS) ACGME Accredited Neurotology Fellowship Program Directory, accessed November 2021.

**Main Outcome Measures:**

Data were collected via CVs, institutional biographies, and emailed questionnaires. Data collected includes age, gender, race and ethnicity, residency and fellowship training institution, time since training completion until FD, length of time as FD, and Hirsch-index (h-index).

**Results:**

Twenty-six FDs were identified, and 17/26 (65.4%) FDs responded to the questionnaire. The majority (23/26; 88.5%) were male. The mean age of male and female FDs was 56 versus 47 years, respectively. Of the 17 that responded to the survey, 82.4% (14/17) self-identified as Caucasian. The mean h-index was 25.4. Older age correlated with a higher h-index (r = 0.46, *P* = 0.019). The duration (mean ± SD, years) from fellowship graduation to FD appointment was 10.7 ± 8.1 and 6.3 ± 4.8 from institutional hire. Six (23.1%) FDs had secondary graduate degrees, and 9 (34.6%) held a leadership position at a national otolaryngology organization.

**Conclusion:**

This observational study assesses demographic data on current neurotology FDs in the United States with an analysis of gender disparities. The objective measures identified can provide a baseline for growth in FD leadership.

Leaders in the sub-specialty practices of medicine, such as neurotology fellowship directors (FD), are responsible for attaining a high level of practice standards and teaching the next generation of surgeons. These individuals in leadership roles share characteristics gained from formal training and mentorship. However, the objective standards that serve as a foundation for selecting neurotology FDs and the characteristics which set them apart from their peers remain unclear. The objective results we seek to identify in this study serve to elucidate trends of current directors within neurotology.

Although there are well-delineated instructions for applicants to both otolaryngology residency and fellowships, the pathway to neurotology leadership positions is not as detailed. Several papers have explored the road map to FD and leader demographic data in other specialties (1–9). Recent literature has only started to examine leadership in otolaryngology, specifically looking at diversity, academic benchmarks, and male versus female representation (10–12). The demographics, objective benchmarks, and common characteristics of neurotology FDs are currently not well-known to the field or to those interested in becoming a FD. This study aims to lay out the career course of current FDs for aspiring FDs and to potentially identify opportunities to progress the FD position, specifically regarding diversifying leaders’ racial, gender, training, and research backgrounds.

## MATERIALS AND METHODS

### Data Collection

We identified neurotology fellowships and their directors from the American Neurotology Society (ANS) Accreditation Council for Graduate Medical Education (ACGME) Accredited Neurotology Fellowship Programs and Directors list, accessed in November 2021 (13). The directory features 26 programs with 26 associated directors. No programs or directors were excluded from this investigation. The directory also provided each institution’s FD name, email, and respective institutional webpage. Credentials of included FDs were confirmed through associated fellowship program webpage. The collection of professional education, residency, residency year, fellowship, and fellowship year was obtained through respective institutional biographies, Doximity (Doximity.com, Doximity Inc., San Francisco, CA), individual curriculum vitaes (CVs), and LinkedIn (Linkedin.com, Sunnyvale, CA). The information was cross referenced between these platforms when applicable. The age of each FD was established using Healthgrades (Healthgrades Operating Company Inc, Denver, CO).

After initial data collection using public resources, emails with a voluntary questionnaire were sent directly to FDs. The email informed the FDs of the study’s objectives with the goal of publication. The voluntary survey was used to record data points that were unavailable online, confirm data found online, and formally request CVs. The demographic information that was of interest included: gender, age, current institution, number of years in the current FD role, name of residency institution and year of graduation, name of fellowship institution and year of graduation, year hired by current institution, year appointed FD, and Scopus h-index. Other data points collected included additional graduate degrees and leadership roles in national otolaryngology societies. Race and ethnicity were self-reported via a free text box in the survey that asked for cultural and ethnic background. Previous leadership papers guided our selection of data points (1,2,5–7). This study did not require approval from the investigational review board (IRB) as determined by the University of Miami IRB.

### Research Productivity and Impact

The scholarly or research productivity and impact level was measured by using each individual’s Scopus h-index, number of publications, and number of citations. The h-index is a numerical value that measures both the productivity and citation impact of an author’s publications. It is calculated by using the highest number of publications that have received at least h citations. For example, an h-index of 10 means that among all publications by the author, 10 of these publications received at least 10 citations each. It is a useful way to quantify the scientific output of a researcher and eliminates outlier publications that may have skewed the impact (14). The h-index and publication count were obtained for each FD by searching the first and last names within the Scopus database (Elsevier B.V., Waltham, MA, USA). All data collected by the lead author were reviewed, verified, and cross referenced to ensure accuracy.

### Statistical Analysis

Descriptive statistics were used to quantify and summarize the data set. H-index data were analyzed using personal correlation coefficients and were interpreted according to the guide of Mukaka for correlation coefficients (15). Pearson correlation coefficients were determined via Statistical Analytics System (Version 9.4 of the SAS System. Copyright © 2013 SAS Institute Inc, Cary, NC). Values under 0.3, 0.3–0.5, 0.5–0.7, 0.7–0.9, and greater than 0.90 indicate a negligible, low, moderate, high, and very high positive correlation, respectively. Statistical significance was considered at an alpha value of *P* < 0.05. Two-sample t-test could not be used to make gender-wise comparisons due to the small female sample size.

## RESULTS

All 26 (100%) FDs in the 2021 ANS Fellowship Directory were included in this study. Twenty-three (88.5%) were male, and 3 (11.5%) were female (Table 1). The mean age of FDs was 55 years (SD 10.2, min 39, max 75). The mean age of male FDs and female FDs was 56 versus 47 years, respectively. The average age of FDs at appointment was 44.2 years (SD 7.4, n = 16). In this study, 17 (65.4%) FDs responded to the survey and all 17 self-reported their race, 14 as white (82.3%), 2 as Asian (11.8%), and 1 as biracial: African American and white (5.9%). Three out of 17 participants self-reported their ethnicity as Hispanic (17.7%) (Table 1).

**TABLE 1.** Demographics including gender, age, and race of neurotology fellowship directors

Gender and age (n = 26)	
Male—n (%)	23 (88.5%)
Female—n (%)	3 (11.5%)
Mean age—n ± SD	54.96 ± 10.12
Median age—n (Min, Max)	56.5 (39, 75)
**Race and ethnicity (n = 17**)	
White	14 (82.4%)
Asian	2 (11.8%)
Black	0 (0%)
Biracial	1 (5.9%)
Hispanic	3 (17.7%)
Non-Hispanic	14 (82.4%)

Gender and age demographic data of all 26 fellowship directors. Race and ethnicity were self-reported by 17 fellowship directors.

The mean duration from fellowship graduation to appointment was 11 years (SD 8.1, min 2, max 31, n = 16). The average length of duration of employment at the FD’s current institution until the time of this study was 16.8 years (SD 10.2, min 4, max 39, n = 17), while the average length of time between hire and FD appointment was 6.3 years (SD 4.8, min 0, max 15, n = 21). The average time from fellowship graduation to FD appointment for males and females was 10.6 and 11.5 years, respectively. The average length of time between institutional hire and FD appointment for males was 6 years and 8.5 years for females. The mean duration in the current FD role was 10.7 years (SD 9.0, min 0, max 31, n = 16)

Institutional affiliation was compared by assessing the FDs’ residency, fellowship, and FD appointment institutions (Table 2). Ten (38.5%) FDs were at the same institution where they completed residency, and 8 (30.8%) FDs were employed at the same institution as their fellowship training. Nine (34.6%) FDs completed training at the same residency and fellowship institutions, and 6 (23.1%) were appointed FD at this same institution.

**TABLE 2.** Education, employment, and leadership characteristics of neurotology fellowship directors

Education and employment	
Calendar year of residency graduation—mean ± SD (n)	1999 ± 10.1 (n = 21)
Duration from residency graduation to earning position of FD—mean ± SD (n)	12.3 ± 7.8 (n = 16)
Calendar year of fellowship graduation—mean ± SD (n)	2001 ± 10.4 (n = 21)
Duration from fellowship graduation to earning position of FD—mean ± SD (n)	10.7 ± 8.1 (n = 16)
Duration of employment at current institution—mean ± SD (n)	16.8 ± 10.2 (n = 17)
Duration that FD has held position as FD—mean ± SD (n)	10.7 ± 9.0 (n = 16)
Time from year of hire to yea3r promoted to FD—mean ± SD (n)	6.3 ± 4.8 (n = 16)
Age of appointment to FD—mean ± SD (n)	44.2 ± 7.4 (n = 16)
**Institution (n = 26**)	
FDs currently working at same institution as residency training—n (%)	10 (38.5%)
FDs currently working at same institution as fellowship training—n (%)	8 (30.8%)
FDs currently working at same institution as both residency and fellowship training—n (%)	6 (23.1%)
FDs who trained at same institution for residency and fellowship—n (%)	9 (34.6%)

Educational and employment progression shows the average and standard deviation (SD) associated with each factor presented. Institution reveals the number of fellowship directors at the same residency, fellowship, or both for which they currently work (n = 26).

**FIG. 1.**
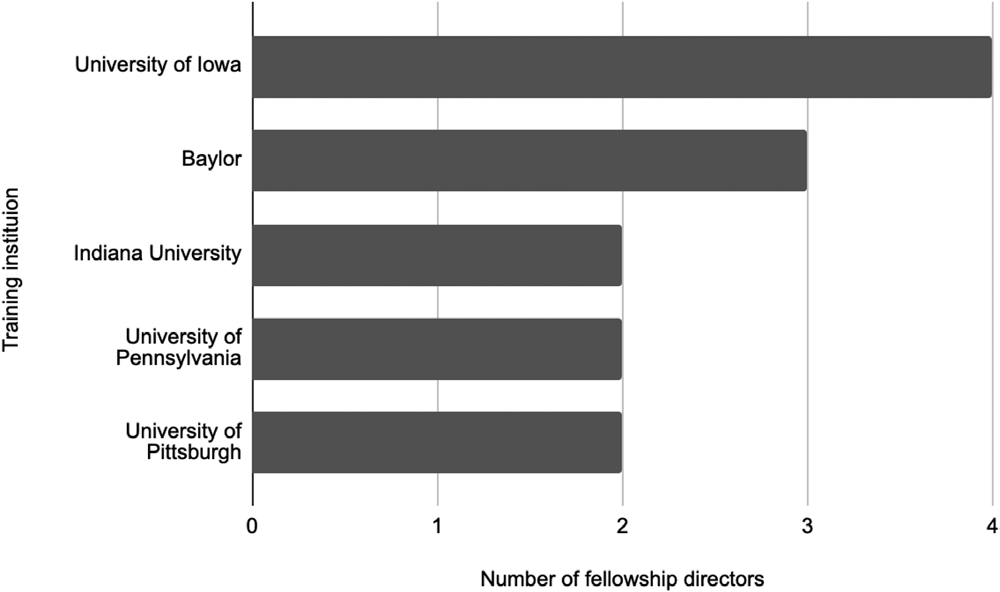
A summary of the most attended residency training programs among current neurotology fellowship directors. Residency programs at which at least 2 fellowship directors trained were included.

**FIG. 2.**
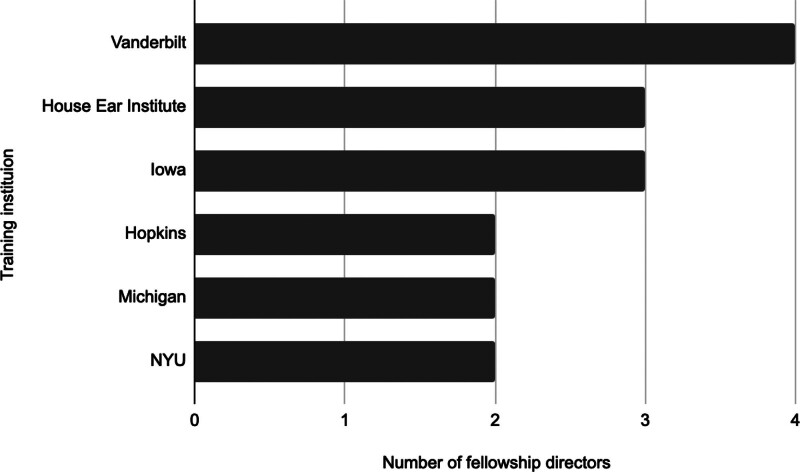
A summary of the most attended fellowship training programs among current neurotology fellowship directors. Fellowship programs at which at least 2 fellowship directors trained were included.

The 5 institutions that the most FDs attended for otolaryngology residency were the University of Iowa (n = 4), Baylor University (n = 3), Indiana University (n = 2), University of Pennsylvania (n = 2), and University of Pittsburgh (n = 2) (Fig. 1). The 3 institutions that the most FDs attended for neurotology fellowship were Vanderbilt (n = 4), followed by House Ear Institute affiliated with the University of Southern California (n = 3), and University of Iowa (n = 3) (Fig. 2).

When looking at leadership in the largest national otolaryngology organizations (AAO, AOS, ABO, ALA, ANS, ARO, TRIO), 12 (46.2%) FDs held a leadership position. The positions included were President, Vice President, Treasurer, and Secretary. Furthermore, 6 of the 26 (23.1%) FDs had an additional degree. Three (11.5%) were PhDs, and 3 (11.5%) had master’s degrees including, Master of Science, Master of Business Administration, and Master of Science in Clinical Investigation.

**FIG. 3.**
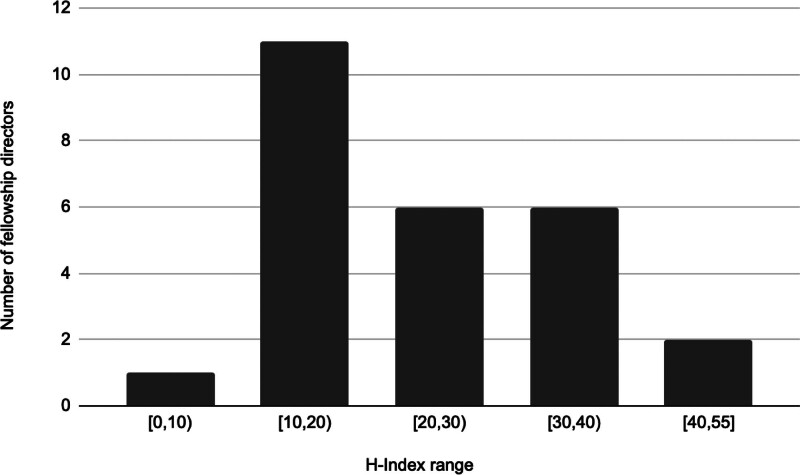
A representation of the Scopus H-indices of all current neurotology fellowship directors. The Scopus h-index values are as of December 1, 2021. Interval notation is used. “[”: Indicates that the range includes the adjacent numerical value “(”: Indicates that the range does not include the adjacent numerical value.

Scholarly performance was assessed using the h-index, number of publications, and number of citations, of which data for all 26 FDs were available (Table 3). The mean number of peer-reviewed publications was 117.1 (SD 76.6, min 23, max 365). The mean number of citations was 2831 (SD 2693, min 235, max 10,639). H-indices ranges were grouped, and the number of total FDs were assessed, including 0–10 (n = 1), 10–20 (n = 11), 20–30 (n = 6), 30–40 (n = 6), and 40–50 (n = 2) (Fig. 3). The overall mean h-index was 25.4 4 ± 11.5. The most impactful FD had an h-index value of 55, and the minimum h-index value was 9. The mean h-index was 26.1 for males and 19.3 for females. Years as FD, age, and residency graduation year were moderately correlated with h-index (Table 3).

**TABLE 3.** Research statistics of neurotology fellowship directors

Research	
Scopus h-index—mean ± SD	25.4 ± 11.6
Scopus h-index—median (Min, Max)	23.5 (9, 55)
Number of publications—mean ± SD	117.1 ± 76.6
Number of publications—median (Min, Max)	101 (23, 365)
Number of citations—mean ± SD	2831 ± 2693
Number of citations—median (Min, Max)	1808 (235, 10639)
**Correlated h-indices**	
Years as FD vs Scopus h-index—r (*P* value)	0.57 (0.021)*
Age vs Scopus h-index—r (*P* value)	0.46 (0.019)*
Residency graduation year vs Scopus h-index—r (*P* value)	0.50 (0.020)*

Research data were publicly available information on SCOPUS for all 26 fellowship directors. H-indices were correlated via Pearson correlation to indicate a relationship between years as fellowship director, age, and time since residency graduation. (*) denotes that a value was statistically significant (*P* < 0.05).

## DISCUSSION

FDs have a valuable impact on the education of trainees and future leaders in their field. Multiple studies have begun to document the qualities of FDs in other fields, but data is lacking in otolaryngology and the respective subspecialties. This analysis of the 26 FDs in neurotology provides a snapshot in time of current academic leaders in the field. It thus should not be seen as an absolute representation of neurotology or otolaryngology.

At the time of our analysis, there were only 3 (11.5%) female FDs. Altogether, women are underrepresented in the field of otolaryngology. The 2020 AAMC Physician Specialty Data Report revealed that there are 1,784 (18.3%) active female otolaryngologists (16). Almost a 50% increase from 2010 when women only represented 12.7% of practicing otolaryngologists (17). However, in 2019, more than one-third (36.3%) of the active physician workforce in the United States was female (16). While otolaryngology has made progress in female representation, further progress can be made, especially when compared with many other specialties. When comparing otolaryngology to surgical fields, neurotology gender representation in leadership is similar to the findings in other surgical subspecialties (5,6). Female representation amongst neurotology FDs is similar to otolaryngology leadership data published in a 2020 study, which evaluated otolaryngology leadership positions and found that 26.5% (27/102) of residency directors, 5.1% (5/99) of chairs, and 14.7% (30/204) of fellowship directors were women (11). Leadership in otolaryngology is not yet representative of otolaryngology residency. The 2021 AAMC report on residents revealed that 37.7% of all otolaryngology residents (international medical school graduates, U.S. and Canadian MD Graduates, U.S. DO Graduates) were females (18). As more women enter otolaryngology residency, this analysis could be used to support initiatives that seek to enlist female members, provide women access to mentorship and research, and encourage women to participate in leadership roles.

There was a notable difference in the gender-wise comparison of FD age. The mean age of females was 47 years, almost 10 years younger than their male colleagues. However, the average age at FD appointment was similar, being 44 for males and 45 for female. Therefore, females are younger on average because they were recently appointed. This demonstrates that leadership demographics are actively changing. We suspect this age gap will close over time as females will be in leadership positions longer.

Clinical service, teaching, and research define academic careers within surgery. Involvement and productivity in research are significant metrics among those who achieve academic leadership positions. Our analysis demonstrates that FDs had a mean h-index of 25.4, with the 5 FDs with the greatest research productivity having h-indices greater than 35. Interestingly, the FDs with the highest h-indices were at institutions that produced the most FDs. Although the h-index does not consider the magnitude or perceptions of the articles published by an author, it has been a useful metric for analyzing an author’s productivity (19,20). However, the h-index is often directly correlated with the papers’ longevity and, thus, the physicians’ career length. One study by Svider et al. found that h-index values of academic otolaryngologists were higher with increased academic rank among the levels of assistant professor, associate professor, and professor (19). The mean age of female FDs was almost 10 years younger than their male colleagues, which could account for their lower mean h-index of 19.3 compared to 26.1 for males.

Like many other leadership roles, experience is crucial to ascending to the FD role. The average duration from residency graduation to FD appointment was 12 years, and the average duration from the year of hire to FD appointment was 6 years. Although we do not have objective data on how FDs previously advanced in their career, our data suggest that mentorship, development, and advancement through the ranks of assistant, associate, and full professor contribute to becoming the FD. In addition, once an FD has attained the position, they tend to stay in the role for a substantial amount of time as demonstrated by a 10 year mean duration in the current role with a maximum of 31 years. Institutional affiliation was assessed, and it appears that training at an institution for residency or fellowship inclines a neurotologist to be FD at that institution as 10 (38.5%) FDs were at the same institution where they completed residency, and 8 (30.8%) FDs were employed at the same institution as their fellowship. Amongst the 26 FDs, there were 18 residency institutions and 16 fellowship institutions where training was completed. Three fellowship programs: Vanderbilt, House Ear Institute, and Iowa, produced 38.5% (10/26) of all the current neurotology FDs. This indicates that specific fellowship training programs may correlate with future academic leadership possibilities.

Race and ethnicity were assessed via a free text survey questionnaire on a volunteer basis. Of the 17 respondents, a majority identified as Caucasian (82.3%). While the current otolaryngology residency class is 63.2% Caucasian, interestingly, the trainees in otology-neurotology were 82.8% Caucasian, as reported by the AAMC (18). As diversity in medicine continues to improve, greater diversity in neurotology could be encouraged via pipeline and research programs that engage medical students and residents from different backgrounds to pursue neurotology. A focus on diversity should remain at the forefront, as it is pivotal for the advancement of a healthcare system to represent the population it serves.

Approximately half of FDs reported leadership roles in otolaryngology and neurotology societies. While society membership is not a requirement for academic leadership, the benefits of the societies include access to collaborative research, networking, relationship building, and society leadership roles. These opportunities provide FDs and aspiring FDs objective returns as well, including scholarships, grants, and educational opportunities. Furthermore, 6 (23.1%) of the FDs had secondary graduate degrees (PhD, MBA, MS), which may provide additional exposure to cutting-edge research, medical device development, and administrative advancement that could aid in the ascent of professorship and leadership.

This study has several limitations, including the small sample size and sensitivity of these results to time. Although we did capture neurotology FDs and could make conclusions within this cohort, it is impossible to generalize these findings to all the otolaryngology subspecialties. We acknowledge that many qualitative factors can impact an individual’s appointment and pursuit of academic leadership positions. For research’s sake and to diminish subjective bias, objective data was solely used for analysis. Despite the use of multiple resources, we did not achieve complete data collection and had partial data for some categories evaluated. With only 3 female FDs, we could not use statistical analysis such as a two-sample t-test to make gender-wise comparisons. These findings are subject to change with time and do not reflect longitudinal trends within leadership but rather set a baseline. It is likely that some of these results have changed since data collection and analysis.

## CONCLUSION

This is the first study to capture current demographic trends and characteristics of neurotology FDs. Although only 11.5% of neurotology FDs were females, we suspect this percentage will continue to rise as more females enter the field of otolaryngology. Neurotology FDs are characterized by their high research output and have significant institutional affiliation. Many FDs had additional graduate degrees and held a leadership position at a national otolaryngology organization. As fellowships grow and diversify, this information will be a useful baseline to measure change over time in FD leadership. We hope this data will encourage aspiring leaders in the field.

## FUNDING SOURCES

None declared.

## CONFLICT OF INTEREST

None declared.

## DATA AVAILABILITY STATEMENT

The datasets generated during and/or analyzed during the current study are not publicly available but are available from the corresponding author on reasonable request.
